# SOD3 and IL-18 Predict the First Kidney Disease-Related Hospitalization or Death during the One-Year Follow-Up Period in Patients with End-Stage Renal Disease

**DOI:** 10.3390/antiox11061198

**Published:** 2022-06-18

**Authors:** Yu-Hsien Liu, Yu-Hsuan Chen, Chi-Hua Ko, Chia-Wen Kuo, Chih-Ching Yen, Wei Chen, Kowit-Yu Chong, Chuan-Mu Chen

**Affiliations:** 1 Department of Life Sciences, Ph.D. Program in Translational Medicine, National Chung Hsing University, Taichung 402, Taiwan; yuhsien000@yahoo.com.tw (Y.-H.L.); yhchen1218@smail.nchu.edu.tw (Y.-H.C.); kochbye@gmail.com (C.-H.K.); kuochiawen@yahoo.com.tw (C.-W.K.); 2 Department of Internal Medicine, Jen-Ai Hospital, Dali Branch, Taichung 402, Taiwan; 3 Division of Rheumatology, Allergy and Immunology, Chang Gung Memorial Hospital, Yunlin 638, Taiwan; 4 Department of Internal Medicine, Taichung Armed Forces General Hospital, Taichung 411, Taiwan; 5 Department of Internal Medicine, China Medical University Hospital, College of Health Care, China Medical University, Taichung 404, Taiwan; d5210@mail.cmuh.org.tw; 6 Division of Pulmonary and Critical Care Medicine, Chia-Yi Christian Hospital, Chiayi 600, Taiwan; peteralfa2004@gmail.com; 7 Department of Medical Biotechnology and Laboratory Science, College of Medicine, Chang Gung University, Taoyuan 333, Taiwan; 8 Hyperbaric Oxygen Medical Research Lab, Bone and Joint Research Center, Chang Gung Memorial Hospital, Linkou, Taoyuan 333, Taiwan; 9 The iEGG and Animal Biotechnology Center, The Rong Hsing Research Center for Translational Medicine, National Chung Hsing University, Taichung 402, Taiwan

**Keywords:** reactive oxygen species, plasma superoxide dismutase 3 (SOD3), interleukin-18 (IL-18), inflammatory cytokines, end-stage renal disease (ESRD), first kidney disease-related hospitalization or mortality

## Abstract

End-stage renal disease (ESRD) patients experience oxidative stress due to excess exogenous or endogenous oxidants and insufficient antioxidants. Hence, oxidative stress and inflammation cause endothelial damage, contributing to vascular dysfunction and atherosclerosis. Therefore, ESRD patients suffer more cardiovascular and hospitalization events than healthy people. This study aims to test the correlations between ROS, SOD3, IL-2, IL-6, and IL-18 and the first kidney disease-related hospitalization or death events in ESRD patients undergoing regular hemodialysis. A total of 212 participants was enrolled, including 45 normal healthy adults and 167 ESRD patients on regular dialysis. Blood samples from all participants were collected for ROS, SOD3, IL-2, IL-6, and IL-18 measurement at the beginning of the study, and every kidney disease-related admission or death was recorded for the next year. Multivariate analysis was conducted by fitting a linear regression model, logistic regression model, and Cox proportional hazards model to estimate the adjusted effects of risk factors, prognostic factors, or predictors on continuous, binary, and survival outcome data. The results showed that plasma SOD3 and serum IL-18 were two strong predictors of the first kidney disease-related hospitalization or death. In the Cox proportional hazards models (run in R), higher IL-18 concentration (>69.054 pg/mL) was associated with a hazard ratio of 3.376 for the first kidney disease-related hospitalization or death (95% CI: 1.2644 to 9.012), while log(SOD3) < 4.723 and dialysis clearance (Kt/V; 1.11 < value < 1.869) had a hazard ratio = 0.2730 (95% CI: 0.1133 to 0.6576) for reducing future kidney disease-related hospitalization or death. Other markers, including body mass index (BMI), transferrin saturation, total iron binding capacity, and sodium and alkaline phosphate, were also found to be significant in our study. These results reveal the new predictors SOD3 and IL-18 for the medical care of end-stage renal disease patients.

## 1. Introduction

Chronic kidney disease (CKD) puts a heavy health burden on society, and its high economic costs include costs for physician visits, ambulatory care, hospitalization, dialysis, and medication [1]. In particular, the cost for end-stage renal disease (ESRD) patients undergoing renal replacement therapy is much higher than that for patients in other CKD stages. CKD is an immune equilibrium disease that leads to a chronic persistent inflammatory state in ESRD patients and high inflammatory effects from high-sensitivity C-reactive protein (hs-CRP), interleukin-6 (IL-6), and tumor necrosis factor-α (TNF-α) in serum [2,3]. IL-6 is a systemic inflammatory cytokine and a predictor of protein-energy wasting atherosclerosis in CKD and ESRD patients. However, no valid predictors of the first kidney disease-related hospitalization or death events in ESRD patients undergoing regular hemodialysis have been identified.

In general, Toll-like receptor (TLR) overexpression in neutrophils and monocytes may enhance cytokine synthesis, with a subsequent inflammatory response. A clinical study recruited 47 CKD, 13 hemodialysis (HD), and 71 control individuals, finding that the elevation of TLR-4 expression in neutrophils was significantly higher in dialysis patients than in stage 3 and 4 CKD patients. Serum levels of TNF-α, IL-6, and monocyte chemoattractant protein-1 (MCP-1) were also significantly increased in HD and CKD patients compared with healthy controls [4]. IL-18 acts as a proinflammatory cytokine and is involved in the primary immune response [5]. One study showed that serum IL-18 has a positive correlation with dialysis age but a negative correlation with dialysis clearance (Kt/V) in predialysis, hemodialysis, and peritoneal dialysis patients [6]. Thus, dialysis efficiency can affect serum IL-18 levels as well as changes in the levels of another inflammatory cytokine, IL-12 [7].

The higher levels of reactive oxygen species (ROS) in uremic patients are due to an imbalance in their production/degradation ratio. This has been validated in patients and models of renal insufficiency, even in early CKD [8]. The plasma total antioxidant capacity (TAC) was observed to be the same in CKD stages 2−4 as under normal renal function; however, TAC fell slightly in ESRD patients [9]. A previous clinical study of 31 HD patients and 18 healthy subjects demonstrated marked increases in oxidative stress markers and decreases in antioxidant defense markers in HD patients [2]. Superoxide dismutase (SOD) is an antioxidant enzyme with three isoforms in mammals, including the cytosolic SOD1, the mitochondrial SOD2, and the extracellular SOD3 [10]. Among them, SOD3 suppresses inflammation by eliminating ROS production, altering immune cell function, inhibiting inflammatory mediators, and regulating cellular signaling cascades (TLR, NF-κB, MAPK, and JAK-STAT) [11].

ESRD patients suffer an excess of morbidity and mortality due to cardiovascular disease (CVD), which cannot be fully explained by the classical CVD risk factors [12]. In addition, elevated CRP, IL-6, and fibrinogen are independent predictors of cardiovascular outcomes in CKD patients [13]. In addition to CVD risk factors, oxidative stress and inflammation are currently emerging as important factors. Infections also play a key role in morbidity and mortality in ESRD patients. Nevertheless, there is little discussion related to oxidative stress and inflammation. In a previous report observing 184 dialysis patients, IL-18 was found to be a strong predictor of hospitalization by multivariate analysis [14]. In another study, both IL-6 and CRP were novel predictors of all-cause and CV mortality, being superior to IL-1, IL-18, TNF-α, ICAM-1, and VCAM-1 [15].

In this study, we aimed to elucidate the correlation between different potential predictors (ROS, SOD3, hs-CRP, IL-2, IL-6, and IL-18) and first nephropathy-related hospitalization or death in ESRD patients receiving hemodialysis.

## 2. Materials and Methods

### 2.1. Study Design

We designed a dual-center prospective study and recruited patients receiving routine hemodialysis at Jen-Ai Hospital or Xinren Clinic (Dali, Taichung, Taiwan). We sampled blood from ESRD patients prior to initiation of dialysis treatment at the start of the study and then tracked and recorded each patient’s clinical status (hospitalization or death) from 1 September 2020 to 30 September 2021. Our primary observed outcome was the first hospitalization or death related to kidney disease. The Ethics Committee of the Clinical Center (ECCC) of Jen-Ai Hospital approved the study (IRB No: 109-76), and all the patients signed the written informed consent.

### 2.2. Participants

**Figure 1:**
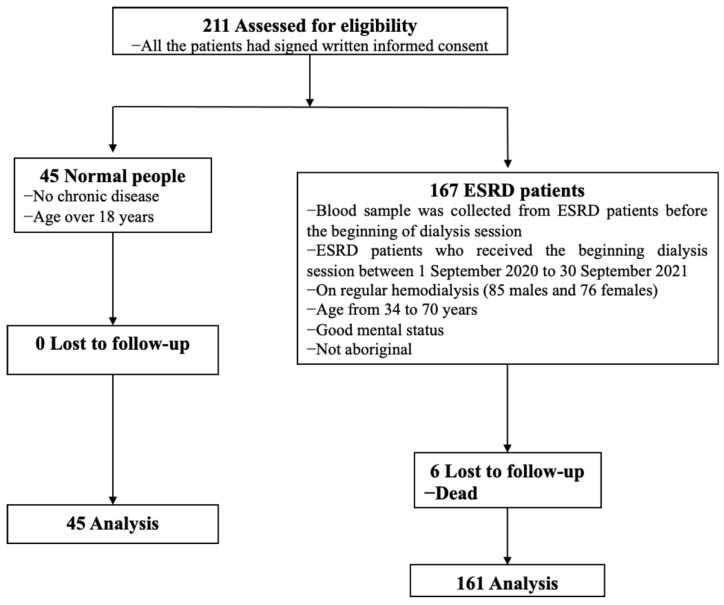
The enrollment flow chart of this study. A total of 211 participants, including 167 ESRD patients receiving regular hemodialysis at Jen-Ai Hospital or Xinren Clinic Hospital, and 45 healthy adults was enrolled. The Ethics Committee of the Clinical Center of Jen-Ai Hospital approved the study, and all the patients signed written informed consent.

A total of 167 ESRD patients was enrolled in this study. The ESRD diagnosis was made from clinical data and symptoms and was approved by the Office of Health Insurance. All patients underwent an average 4 h hemodialysis program 3 times per week. The exclusion criteria were as follows: age under 30 or over 70 years old, malignancy, acquired immunodeficiency, indigenous people, and those taking antioxidants. We also collected the relevant data from all patients, including age, sex, BMI, primary kidney disease, complete blood count (CBC), monthly routine biochemical test data, Kt/V, Fe supplement dose, serum hs-CRP, IL-2, IL-6, and IL-18. During the 1-year follow-up period, we recorded every causal hospitalization or death related to kidney disease. The enrollment flow chart of this study is shown in Figure 1.

### 2.3. Oxidative Stress, Inflammatory Cytokines, and SOD Activity Assays

Blood samples from normal adults and dialysis patients were collected in CAT blood collection tubes (VACUETTE, Cat. No. 454098, Austria) for ROS/RNS and inflammatory cytokine assays and in LH Lithium Heparin tubes (VACUETTE, cat. No. 454237, Kremsmünster, Austria) for SOD determination. Each blood sample was centrifuged at 10,000 rpm for 10 min at 4 °C, and the supernatant serum was transferred to a new tube. One hundred microliters of serum was subjected to the ROS/RNS assay (OxiSelect kit, Cat. No. STA-347, Cell Biolabs, San Diego, CA, USA), and the fluorescence content was measured on a multimode microplate reader (Molecular Devices, San Jose, CA, USA). SOD3 activity was measured in 50 µL of plasma (Human SOD3/EC-SOD ELISA kit, Cat. No. ab277415, Abcam, Waltham, MA, USA) [16,17]. Serum in a total volume of 50 µL was assayed for inflammatory cytokines, including IL-2, IL-6, and IL-18 (BD^TM^ Human Cytokine/Chemokine Panel A, Cytometric Bead Array, Cat. No. 558267, BD Biosciences, San Jose, CA, USA).

### 2.4. Statistical Analysis

Statistical analysis was conducted using R 4.1.2 software (R Foundation for Statistical Computing, Vienna, Austria) as previously described [18]. In statistical testing, a two-sided *p* value ≤ 0.05 was considered statistically significant. Continuous variables were expressed as mean ± standard deviation (SD), categorical variables were presented as frequency and percentage (%), and the survival curves of time to the first kidney disease-related hospitalization or death were estimated by the Kaplan−Meier method. In the univariate analysis, the unadjusted effect of each potential risk factor, prognostic factor, or predictor of the first-time kidney disease-related hospitalization or death was examined using the Wilcoxon rank-sum test, Fisher’s exact test, chi-squared test, and log-rank test, as appropriate for the type of data. Next, a multivariate analysis was performed to estimate the adjusted effects of risk factors, prognostic factors, or predictors on the time to the first kidney disease-related hospitalization or death by fitting a Cox proportional hazards model.

The regression analysis procedure followed the recommended method as previously described [18,19]. Briefly, to find a parsimonious regression model suitable for our data for effect and estimation outcome prediction, well-known model fitting techniques were used, including variable selection, goodness of fit (GOF) assessment, and regression diagnostics and remedies. Precisely, we applied the stepwise variable selection procedure to obtain the best candidate final regression model using the My Stepwise package [20]. All the significant and non-significant relevant covariates in univariate analysis listed in Table 1 and some of their interaction terms or moderators were input into the multivariable regression. The significance levels for entry (SLE) and staying (SLS) were set at 0.15 to be conservative. Then, with substantial knowledge and clinical experience, the best candidate final regression model was manually determined by manually removing covariates with a *p* value > 0.05 until all regression coefficients had effects significantly different from 0 [19].

The GOF measures were examined, including concordance and adjusted generalized *R*^2^, to assess the GOF fitted to the Cox proportional hazards model. The concordance, which is a value between 0 and 1 in the Cox proportional hazards model, is equivalent to the c statistic in logistic regression, so a value ≥ 0.7 suggests an acceptable level of discrimination power. However, the values of adjusted generalized *R*^2^ (0 ≤ *R*^2^ ≤ 1), proposed by Van der Net [21], are usually low for Cox proportional hazards models—in our experience, adjusted generalized *R*^2^ ≥ 0.15 indicates an acceptable fit for a Cox proportional hazards model.

## 3. Results

### 3.1. Patient Characteristics

A total of 161 adult ESRD patients receiving hemodialysis (85 males and 76 females) was enrolled for analysis. Patient characteristics, including sex, age, body mass index (BMI), etiology of primary renal disease, basic monthly biochemical data, ROS, SOD3, IL-2, IL-6, and IL-18, were analyzed (Table 1). Patient age ranged from 34 to 70 years (average: 59.11 ± 8.43 years). The primary renal disease originated from diabetes (DM, 49.7%), chronic glomerulonephritis (CGN, 28.6%), hypertension (16.8%), and other diseases (5%). Diabetes patients had a higher tendency for first kidney disease-related hospitalization or death (*p* < 0.005) than other primary renal diseases. As shown in Table 1, the level of HbA1c in DM subgroup of ESRD patients with hospitalization was slightly increased (7.30 ± 1.33%) compared to the nonhospitalization group (6.82 ± 1.19%); however, there were no significant difference (*p* = 0.4761). Serum alkaline phosphate had more significant expression in the hospitalization group (81.92 ± 34.22 IU/L) than the nonhospitalization group (66.87 ± 22.71 IU/L; *p* = 0.005). Other variables, including RBC number, albumin, creatinine, Fe, total iron binding capacity (TIBC) and IL-6, were significant according to the primary statistics (*p* < 0.005). Hospitalization frequency and hospitalization days within 1 year before the start of the study were also meaningful. On the topic of nutrition, serum albumin was significantly different between the nonhospitalization (4.37 ± 0.53 mg/dL) and hospitalization (4.20 ± 0.50 mg/dL, *p* < 0.05) groups by the Wilcoxon rank-sum test for continuous variables and Fisher’s exact test for categorical variables, but BMI was similar. Of the proinflammatory cytokines, only IL-6 was significant, and IL-2 and IL-18 were not significant, by univariate analysis.

### 3.2. Total Hospitalization Events Prior to Study Initiation Was Positively Linearly Associated with Further First Kidney Disease-Related Hospitalization or Death

**Figure 2:**
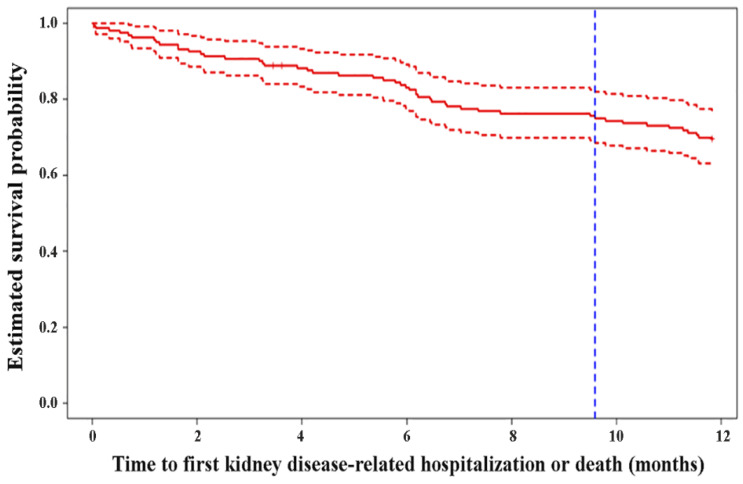
Kaplan−Meier estimate of survival curve of time to the first kidney disease-related hospitalization or death. The 75th percentile of survival time was approximately 9.5 months.

**Figure 3:**
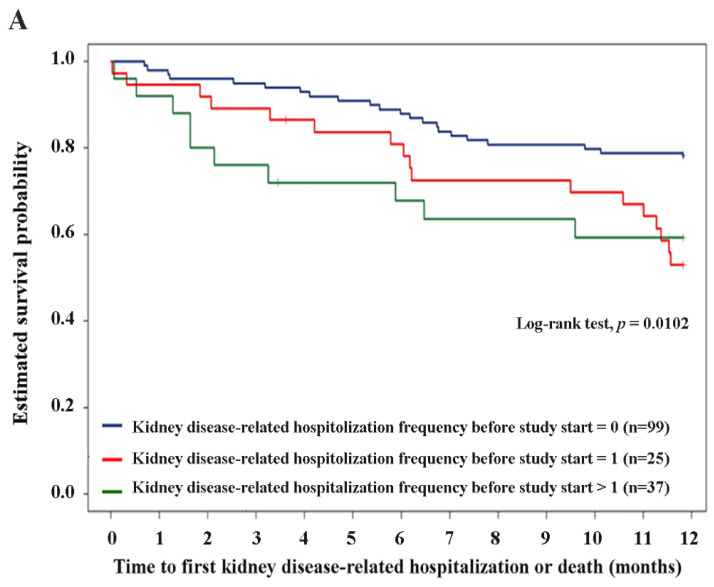

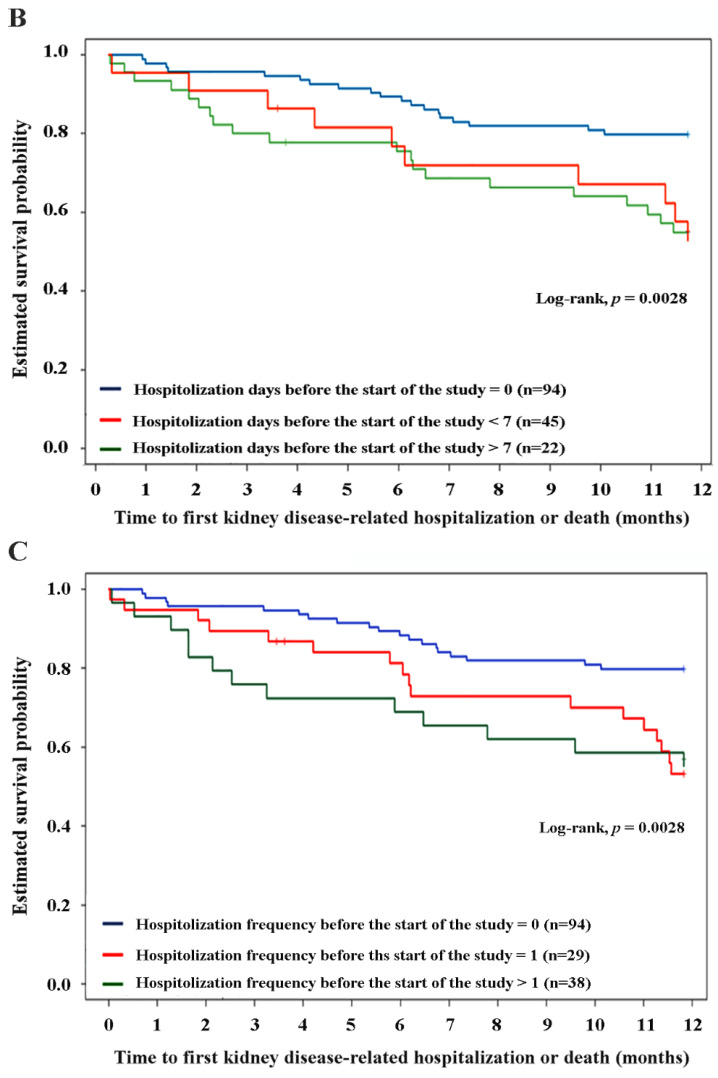
Kaplan−Meier estimate of the survival curve for time to the first kidney disease-related hospitalization or death stratified by (**A**) all-cause hospitalization frequency, (**B**) all-cause hospitalization days, and (**C**) kidney disease-related hospitalization frequency. (**A**–**C**): Hospitalizations were all within one year before the start of the study.

**Figure 4:**
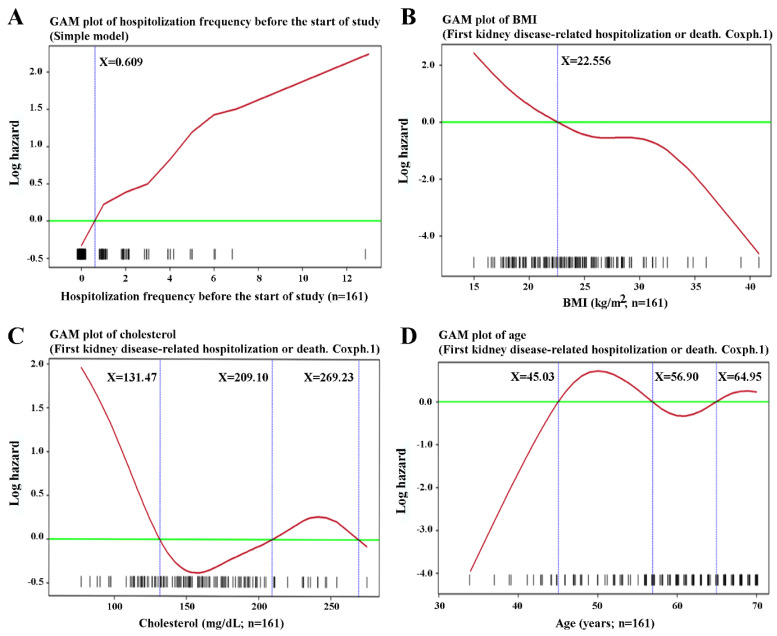
Generalized additive model (GAM) plots of the first kidney disease-related hospitalization or death versus (**A**) hospitalization frequency within 1 year before the start of the study (0.3345 ± 0.0741, *p* < 0.0001), especially those with a level over 0.609; (**B**) BMI (kg/m^2^) (1.1039 ± 0.3986, *p* = 0.0056), high risk if BMI < 22.556; (**C**) cholesterol (mg/dL); and (**D**) age (years) (1.2961 ± 0.3834, *p* = 0.0007), high risk if age over 45.027 years and under 56.9 years.

Figure 2 shows the estimated survival probability due to kidney disease-related hospitalization or death, as calculated by Kaplan–Meier survival analysis. The 75th percentile of survival time was 9.59 months. We also found a shorter estimated survival probability among patients with hospitalization within 1 year before the start of the study. Specifically, hospitalization frequency (0.598 ± 1.018 vs. 1.714 ± 2.466, *p* = 0.0004), hospitalization days (1.973 ± 3.840 vs. 3.898 ± 4.849, *p* = 0.0011), and kidney disease-related hospitalization frequency (0.571 ± 1.011 vs. 1.653 ± 2.496, *p* = 0.0014) were associated with survival (Table 1 and Figure 3). When multivariate analysis using Cox proportional hazards models in R was applied to the overall study population to adjust for time to first kidney disease-related hospitalization or death according to age, BMI, comorbidity, hs-CRP, electrolyte, lipid status, etc., only the hospitalization frequency within 1 year before the start of the study was significant (hazard ratio: 1.3972, *p* < 0.0001, CI: 1.2084–1.6156) (Table 2). As shown in Figure 4, the high-hospitalization-frequency group (*p* > 0.609) might have been in a more severe chronic inflammatory state, with a high incidence of further kidney disease-related hospitalization or death.

### 3.3. Various Biochemical Parameters Predict First Kidney Disease-Related Hospitalization or Death

**Figure 5:**
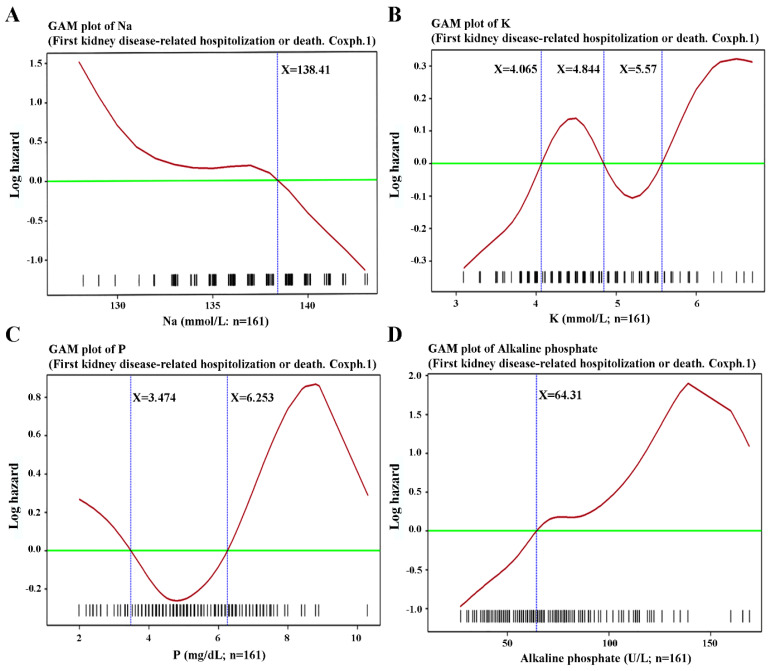
Generalized additive model (GAM) plots of time to first kidney disease-related hospitalization or death versus (**A**) sodium (Na; mmol/L), (**B**) potassium (K; mmol/L), (**C**) phosphate (P; mg/dL), and (**D**) alkaline phosphate (U/L).

**Figure 6:**
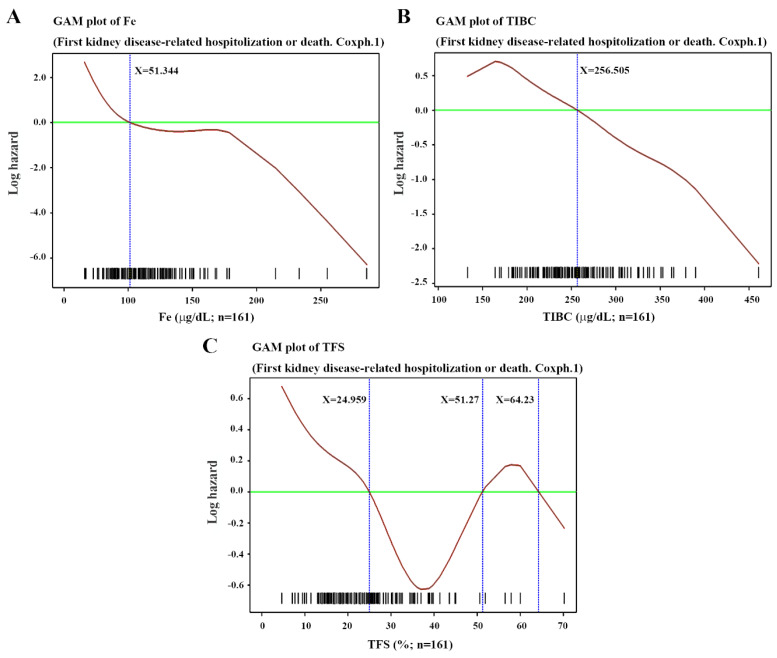
Generalized additive model (GAM) plots of time to first kidney disease-related hospitalization or death versus (**A**) serum iron (µg/dL); (**B**) total iron binding capacity (TIBC; µg/dL), TIBC level below 256.505 µg/dL having a poor outcome; and (**C**) transferrin saturation (TFS; %), TFS < 24.959% or >51.27% bringing high risk.

As shown in Table 2 and Figure 5 and Figure 6, alkaline phosphate > 64.31 U/L, ALT > 15.316 U/L, total iron binding capacity (µg/dL), transferrin saturation < 24.959% or > 51.27%, Na < 139.52 mmol/L, phosphate < 3.747 mg/dL or > 6.253 mg/dL, and cholesterol < 130.054 mg/dL or > 209.364 mg/dL were positive predictors for kidney disease-related hospitalization or death. Age < 45.02 years, or > 56.89 years, and BMI > 22.556 kg/m^2^ also were meaningful (Figure 4). In patients with ESRD, a strange phenomenon that we called the “obesity paradox” or “reverse epidemiology” was consistently reported, i.e., a higher BMI was paradoxically associated with better survival [22]. Serum albumin is also a crucial factor related to mortality and hospitalization in ESRD patients. In other studies, measuring classic parameters of nutritional status has shown that wasting is a poor nutritional sign commonly seen in ESRD patients. In clinical practice, protein-energy wasting (PEW) is often observed in ESRD patients and is caused by a chronic active inflammatory process. With the activation of inflammatory cytokines, such as IL-6 or TNF-α, loss of appetite with subsequent muscle wasting and hypoalbuminemia may be seen [22,23]. In our study, IL-6 (5.1358 ± 8.3857 pg/mL vs. 7.0675 ± 7.7850 pg/mL, *p* < 0.05), hemoglobin (10.18 ± 1.61 g/dL vs. 9.57 ± 1.11 g/dL), and albumin (4.37 ± 0.53 gm/dL vs. 4.20 ± 0.50 gm/dL, *p* < 0.05) were meaningful in the primary statistical results but failed to reach significance in the final Cox proportional hazards model.

### 3.4. Increased IL-18 Concentration Is Associated with a Higher Risk of Future Hospitalization or Death

As shown in Table 1, there was no significant difference between patients who had suffered at least one hospitalization and those who had not (34.71 ± 26.24 vs. 34.35 ± 25.82 pg/mL, respectively, *p* = 0.8629) in the mean serum concentration of IL-18 at baseline by univariate analysis. However, after Cox proportional hazards models were applied to the whole study population, adjusted for first kidney disease-related hospitalization or death according to age, electrolytes, BMI, comorbidity, lipid status, serum iron, nutritional state (albumin), dialysis clearance (Kt/V), hs-CRP, IL-2, and IL-6, serum IL-18 concentration was positively correlated with the time to first hospitalization or death (1.2166 ± 0.5010, *p* < 0.05, hazard ratio: 3.3757, CI: 1.2644–9.0122) in multivariate analysis. Because the pathophysiological events leading to kidney disease-related hospitalization or death included cardiovascular and infectious diseases, serum IL-18 concentration could act as an independent predictor for all-cause hospital admission or death better than other inflammatory mediators. In the study, after multivariate analysis, hs-CRP, IL-2, and IL-6 failed to show significant results.

### 3.5. Plasma logSOD3 Combined with Dialysis Clearance (Kt/V) as a New Predictor of First Kidney Disease-Related Hospitalization or Death

**Figure 7:**
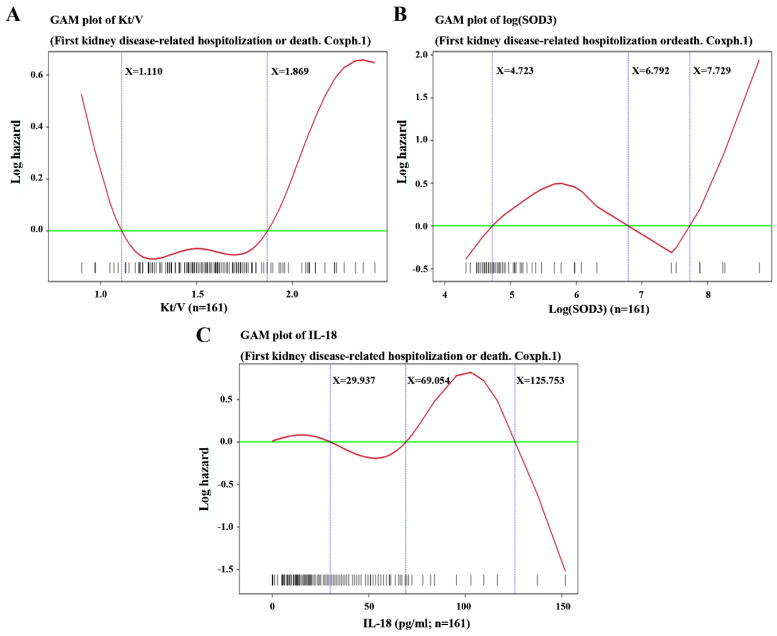
Generalized additive model (GAM) plots of the first kidney disease-related hospitalization or death versus (**A**) Kt/V, where Kt/V below 1.088 or over 1.992 brought a high risk; (**B**) log(SOD3), where log(SOD3) > 4.723 brought a high risk of events. Plasma SOD3 and Kt/V urea are closely related in the clinic. Given a log(SOD3) < 4.723 and Kt/V > 1.11 or <1.869, the patient has a lower risk of kidney disease-related hospitalization or death; and (**C**) IL-18 (pg/mL) (1.2166 ± 0.5010, *p* = 0.0152), where IL-18 over 69.054 conferred a high risk of events.

A negative correlation between plasma logSOD3 and dialysis clearance (Kt/V) in ESRD patients was a statistically significant predicator of first kidney disease-related hospitalization or death events. Increased levels of plasma SOD3 have been observed in many diseases. In a previous study, a rise in the plasma SOD3 level and a decline in renal function were closely correlated in chronic kidney disease patients [24]. In this study, the combination of logSOD3 under 4.723 and Kt/V over 1.11 but under 1.87 had a negative correlation with the outcome of first kidney disease-related hospitalization or death (−1.2982 ± 0.4485, *p* < 0.005, CI: 0.1133–0.6576) (Table 2 and Figure 7).

## 4. Discussion

The objective of our study was to evaluate whether serum markers of inflammation (hs-CRP, IL-2, IL-6, and IL-18), oxidative stress (ROS), and plasma antioxidation (SOD3) were associated with the risk of a first kidney disease-related admission or death event in ESRD patients by the 1 year clinical follow-up. Our statistical calculations showed that there were many significant factors in the results, including hospitalization frequency within 1 year before the study, age, BMI, platelets, ALT, phosphate, alkaline phosphate, sodium, serum iron, TIBC, cholesterol, IL-18, and logSOD3 with Kt/V. Most of these predictors have been discussed in many previous studies. In this study, we were more interested in the roles of SOD3 as an antioxidant and IL-18 as an inflammatory mediator in the disease process of ESRD patients. It is well known that CKD patients are consistently in a low-grade inflammatory state, with increased inflammatory markers CRP, IL-6, TNF-α, and fibrinogen [3,25]. Inflammation is a redox-sensitive mechanism stimulated by oxidative stress that activates the transcription factor NF-κB, which mainly regulates the expression of inflammatory mediator genes, resulting in the production of proinflammatory cytokines [26]. From a different perspective, patients with chronic inflammatory and infectious diseases have a high incidence of coronary artery disease. Memon et al. [27] demonstrated that bacterial lipopolysaccharide (LPS)-induced infection and zymosan-induced inflammation can increase lipoprotein oxidation in animals. Another retrospective study of 96 ESRD patients showed a strong association between high oxidized LDL (ox-LDL) and systolic cardiac dysfunction in ESRD patients treated with permanent hemodiafiltration therapy [28]. Nguyen-Khoa et al. [29] revealed that inflammatory processes increase the production of ROS to deplete antioxidants, followed by increases in lipid peroxidation products and ox-LDL. Interestingly, serum pentraxin-3 and hs-CRP, as markers of inflammation, and serum malondialdehyde (MDA), as a marker of oxidative stress, have been elevated and have shown statistical significance even during a single brief hemodialysis session in ESRD patients [30]. Therefore, inflammation and oxidative stress coexist in dialysis patients, a phenomenon that has adverse effects on the morbidity and mortality of ESRD patients.

Even with new treatment and intervention strategies, the poor outcomes of maintenance hemodialysis in ESRD patients continue to attract worldwide attention. Oxidative stress and chronic inflammation play the most important roles in the long-term complications in HD patients, such as infection, cardiovascular disease, atherosclerosis, anemia, and malnutrition [31,32,33]. Among these proinflammatory cytokines, IL-18 is considered a host defense protein against bacterial, fungal, and protozoan infections and has been shown to be overexpressed in autoimmune and inflammatory diseases, including inflammatory arthritis, insulin-dependent diabetes mellitus (IDDM), multiple sclerosis (MS), inflammatory bowel disease (IBD), pulmonary disease, and other inflammatory diseases [34]. ESRD patients receiving maintenance dialysis treatment have an abnormal physiological condition due to factors such as uremic toxins, anemia, inflammation, volume overload, and hypertension. In peripheral blood mononuclear cells (PBMCs) isolated from CKD and HD patients, NOD-like receptor 3 (NLRP3), apoptosis-associated speck-like protein containing a CARD domain (ASC), inflammasome components (e.g., CASP-1), and the proinflammatory cytokines IL-1β and IL-18 have been found to be abundantly expressed [35]. The inflammasome–caspase-1–IL-1/IL-18 axis is a relatively newly discovered innate immune pathway that is activated in response to infection and deleterious injury [36]. In a previous report on 184 dialysis patients, IL-18, age, and albumin were identified as strong predictors of hospitalization by multivariate analysis [14]. However, CRP and BMI were not included in the results. In our study, IL-18 showed a strong positive linear correlation with all-cause kidney disease-related hospitalization or death. This result is consistent with other studies showing that IL-18 is a strong predictor of cardiovascular events or hospitalization events [14,37,38]. Interestingly, hs-CRP and IL-6 did not show any positive correlation in our Cox proportional hazards model. In two earlier studies of dialysis patients, the authors added IL-6 and CRP to multivariate Cox models of cardiovascular and all-cause death. IL-6 had almost double the effect of CRP [5,15]. In a recent study, the role of IL-6 was more associated with coronary artery calcification and cardiovascular diseases in ESRD patients [39,40]. Although both IL-6 and IL-18 are proinflammatory cytokines, their pathogenesis in clinical disease differs. In our study, we counted hospitalization or deaths from cardiovascular, infectious, and other causes. Therefore, IL-18 is more important than IL-6 in these outcomes.

The expression of SOD3 is extraordinarily complex and ambiguous. SOD3 is a secreted glycoprotein with a unique functional heparin-binding domain (HBD) that is primarily located in extracellular fluids, where it protects cells from damage. Approximately 99% of SOD3 is bound to heparan sulfate proteoglycans in the vascular wall, and the remaining 1% of SOD3 is circulated in equilibrium between the plasma phase and the endothelial glycocalyx [24,41]. Molecular genetic studies have shown that the nucleotide substitution of arginine 213 with glycine in the HBD of SOD3 is associated with significantly higher plasma SOD3 concentrations [42,43]. How plasma SOD3 levels are regulated under normal physiological conditions is unknown. Therefore, elevated plasma SOD3 levels may be the result of a diseases or genetic defects.

In a previous study analyzing plasma SOD3 levels in 504 random blood donors, a common phenotypic variant (2.2%) was linked to a 10-fold increase in plasma SOD3 levels [42]. As for SOD3 levels in CKD, in a study of 185 chronic glomerular disease and 20 HD patients, both plasma bioactivity and plasma Cu/Zn-SOD (SOD3) levels were found to be elevated in chronic glomerular disease patients. However, in patients with ischemic heart disease (IHD), the increase in plasma Cu/Zn-SOD levels was much greater than the increase in SOD bioactivity. This was due to increased monomeric inactivation of Cu/Zn-SOD enzymes [44].

It is well known that ROS, induced by endogenous or exogenous stimuli, play a critical role in the activation of the apoptotic process via mitochondria-dependent and mitochondria-independent pathways [45]. In the face of oxidative stress, cells respond by overexpressing antioxidant genes (SOD1, SOD2, SOD3, and CAT) to counteract the oxidant overcharges [46]. Among them, SOD3 is an extracellular antioxidant that protects cells from oxidative damage. In SH-SY5Y cells, overexpressing SOD3 promoted the survival of the cells by (1) decreasing ROS production, MDA levels, calcium levels, and cytochrome C, caspase-3, caspase-9, and Bax gene expression; and (2) by increasing the gene expression and activity of antioxidant enzyme genes and the expression level of Bcl-2 [47]. Hence, SOD3 has a pivotal role in protecting cells against oxidative damage by its direct antioxidant effects and its indirect induction of antioxidant gene expression. A clinical study in patients with type 2 diabetes has shown significant positive relationships between serum SOD3 concentration and the duration of diabetes, carotid artery intimal−media thickness, the severity of nephropathy, and the severity of retinopathy. The severity of micro- and macrovascular complications in diabetic patients has been closely correlated with serum SOD3 levels [48]. These discoveries suggest that serum SOD3 level might be a factor in vascular injury. The phenomenon of persistent hyperglycemia may induce oxidative stress by damaging the vascular endothelium and decreasing the binding of SOD3 to the vascular wall due to nonenzymatic glycation of SOD3. This pathogenesis partially explains the higher plasma SOD3 levels in various diseases. In our previous study, the serum and urinary SOD3 levels in three different groups of patients (diabetes alone, early CKD, and diabetes with CKD) were measured. Higher serum and urinary SOD3 levels were found in the groups of early CKD and diabetes with CKD than in the diabetes-alone patients [49]. In the kidney, SOD3 expression is mainly detected in the glomerular capillary, arterial/arteriolar wall, and renal tubules [48]. Elevated plasma SOD3 may reflect the change in the expression of SOD3 or SOD3 tissue binding. Therefore, plasma SOD3 could be a marker for vascular injury, revealing the extent of oxidative injury to the vascular endothelium. Another study of 132 CKD patients also found that the plasma SOD3 level was positively correlated with MDA and ox-LDL antibody concentrations [50]. Taken together, the data suggest that ESRD patients with high plasma SOD3 levels may have a highly oxidative and inflammatory status.

Increased oxidative stress is a core event of renal function deterioration in CKD patients. Hence, a reduction in oxidative stress can improve renal function and lower inflammation and CVD risks [51]. Authors have long focused on reducing oxidative stress in patients with chronic kidney disease. Administration of antioxidants, therapeutic substances, and biocompatible membranes can be used as interventions to eliminate oxidative stress in dialysis patients [52]. For example, vitamin E has been shown to reduce serum CRP and monocyte IL-6 levels in type 2 diabetic patients, and vitamin E has been shown to play a more important role than vitamin C in improving oxidant/antioxidant imbalance in HD patients [53].

In this study, plasma SOD3 and IL-18 levels were significantly correlated with the onset of first kidney disease-related hospitalization or death and could be applied as novel predictors in clinical practice. In many studies, the change in plasma SOD3 levels was not consistent between patients at the time of diagnosis. We speculated that the activation of leukocytes and the release of proinflammatory factors in various diseases stimulate the production of ROS, which subsequently promotes the expression of SOD3. In a case−controlled study of 100 patients with coronary artery disease (CAD) and 50 healthy adults, a nonsignificant positive association between MDA and plasma SOD3 was observed [54]. That study concluded that antioxidant levels increase in the initial stages of CAD to protect against oxidative stress, but SOD3 levels decrease as the disease progresses. According to these results, plasma SOD3 levels may have different clinical implications at different stages of various diseases. In the future, it is worth examining these molecules at different time points to paint a complete picture of how they are expressed during the ESRD disease process.

## 5. Conclusions

ESRD patients suffer a higher morbidity and mortality, especially from cardiovascular disease and infections, than the general population. This study discovered that plasma SOD3 and serum IL-18 are better and more efficient predictors of all-cause morbidity and mortality than the common inflammatory mediators IL-6 and hs-CRP in ESRD patients.

## Figures and Tables

**Table 1:** Comparisons of demographic and clinical characteristics between ESRD patients without kidney disease-related hospitalization or death and those with a first kidney disease-related hospitalization or death during the follow-up period.

Variable ^1^	All Patients	Without Kidney Disease-Related Hospitalization or Death = 0	With First Kidney Disease-Related Hospitalization or Death ≥ 1	*p* Value ^1^
Number of subjects (*n*)	161 (100%)	112 (69.5%)	49 (30.5%)	
Age (years)	59.11 ± 8.43	58.79 ± 8.77	59.82 ± 7.63	0.6602
Sex				0.3249
Female	76 (47.2%)	50 (65.8%)	26 (34.2%)	
Male	85 (52.8%)	62 (72.9%)	23 (27.1%)	
Etiology of primary renal disease				
Diabetes mellitus (DM)	80 (49.7%)	56 (70.0%)	24 (30.0%)	0.0037
Hypertension	27 (16.8%)	21 (77.8%)	6 (22.2%)	0.3093
Chronic glomerulonephritis	46 (28.6%)	31 (67.4%)	15 (32.6%)	0.7045
Others	8 (5.0%)	4 (50.0%)	4 (50.0%)	0.2173
Body mass index (kg/m^2^)	23.67 ± 4.51	24.06 ± 4.69	22.77 ± 3.97	0.1368
WBC × 1000 (µL)	6.67 ± 2.01	6.53 ± 2.01	7.00 ± 2.00	0.1343
RBC × 10^6^ (µL)	3.41 ± 0.58	3.48 ± 0.63	3.23 ± 0.39	0.0395
Hb (g/dL)	10.00 ± 1.50	10.18 ± 1.61	9.57 ± 1.11	0.0395
Hct (%)	30.92 ± 4.40	31.50 ± 4.72	29.62 ± 3.25	0.0235
MCV (fL)	91.37 ± 6.72	91.05 ± 7.20	92.12 ± 5.45	0.8270
Platelet × 1000 (µL)	219.87 ± 65.42	223.39 ± 65.18	211.82 ± 65.92	0.4817
Albumin (gm/dL)	4.32 ± 0.52	4.37 ± 0.53	4.20 ± 0.50	0.0381
AST or GOT (IU/L)	16.93 ± 6.36	16.69 ± 6.09	17.47 ± 6.98	0.6231
ALT or GPT (IU/L)	14.97 ± 8.56	14.52 ± 8.59	16.00 ± 8.47	0.1424
Alkaline P (IU/L)	70.75 ± 27.64	66.87 ± 22.71	81.92 ± 34.22	0.0050
Total protein (mg/dL)	6.95 ± 0.70	6.95 ± 0.71	6.94 ± 0.70	0.9648
Cholesterol (mg/dL)	159.33 ± 37.31	160.90 ± 36.35	155.73 ± 39.59	0.4669
Triglyceride (mg/dL)	149.22 ± 101.03	149.58 ± 95.05	148.41 ± 114.58	0.5591
HbA1c (%) in DM	6.97 ± 1.25	6.82 ± 1.19	7.30 ± 1.33	0.4761
Uric acid (mg/dL)	6.53 ± 1.86	6.71 ± 1.84	6.11 ± 1.86	0.1352
Creatinine (mg/dL)	10.10 ± 2.23	10.28 ± 2.11	9.67 ± 2.45	0.0481
BUN (mg/dL)				
Before dialysis	72.34 ± 16.72	71.64 ± 15.35	73.93 ±19.58	0.5418
After dialysis	16.78 ± 5.70	16.74 ± 5.26	16.88 ± 6.64	0.7698
Na (mmol/L)	136.94 ± 2.85	137.20 ± 2.86	136.33 ± 2.76	0.0630
K (mmol/L)	4.61 ± 0.74	4.59 ± 0.72	4.67 ± 0.78	0.5926
Ca (mg/dL)	9.12 ± 0.73	9.15 ± 0.71	9.05 ± 0.78	0.2962
P (mg/dL)	5.241 ± 1.537	5.131 ± 1.448	5.493 ± 1.713	0.2009
Ca × P (mg^2^/dL^2^)	47.79 ± 14.39	46.95 ± 13.70	49.711 ± 15.83	0.3097
Transferrin saturation (%)	24.72 ± 10.56	25.46 ± 10.68	23.02 ± 10.17	0.1129
Ferritin (ng/mL)	477.93 ± 763.61	452.41 ± 801.28	536.24 ± 673.77	0.5298
TIBC (µg/dL)	254.14 ± 47.20	259.63 ± 49.16	241.59 ± 40.10	0.0250
Fe (µg/dL)	64.42 ± 31.02	65.66 ± 33.40	55.02 ± 23.39	0.0431
I-PTH (pg/mL)	288.56 ± 401.61	258.50 ± 276.38	357.27 ± 595.05	0.7243
Kt/V_urea_	1.59 ± 0.30	1.57 ± 0.29	1.61 ± 0.33	0.5615
hs-CRP (mg/dL)	0.72 ± 1.33	0.65 ± 1.25	0.90 ± 1.49	0.1032
IL-2 (pg/mL)	0.1763 ± 0.1737	0.1858 ± 0.1993	0.1548 ± 0.0901	0.8451
IL-6 (pg/mL)	5.7237 ± 8.2317	5.1358 ± 8.3857	7.0675 ± 7.7850	0.0181
IL-18 (pg/mL)	34.46 ± 25.87	34.35 ± 25.82	34.71 ± 26.24	0.8629
ROS (mmol/L)	2102.3 ± 765.5	2127.4 ± 842.5	2044.9 ± 553.8	0.9224
Plasma SOD3 (ng/mL)	260.52 ± 725.67	205.14 ± 480.47	387.12 ± 1094.51	0.1641
log(plasma SOD3)	4.885 ± 0.740	4.832 ± 0.642	5.006 ± 0.924	0.1641
Hospitalization days ^2^	2.559 ± 4.251	1.973 ± 3.840	3.898 ± 4.849	0.0011
Hospitalization frequency ^2^	0.938 ± 1.676	0.598 ± 1.018	1.714 ± 2.466	0.0004
Kidney-related hospitalization frequency ^2^	0.901 ± 1.682	0.571 ± 1.011	1.653 ± 2.496	0.0014

Abbreviations: BUN = blood urea nitrogen, DM = diabetes mellitus, Hb = hemoglobin, HbA1c = glycated hemoglobin, hs-CRP = high sensitivity C-reactive protein, IL = interleukin, I-PTH = intact parathyroid hormone, ROS = reactive oxygen species, SOD = superoxide dismutase, and TIBC = total iron binding capacity. ^1^ The sample statistics presented in this table are mean ±standard deviation (SD) for continuous variables and frequency (percentage, %) for categorical variables. The listed *p*-values of statistical tests were calculated using the Wilcoxon rank-sum test for continuous variables and the chi-squared test for categorical variables. ^2^ Hospitalization occurred within the 12 months before blood sampling.

**Table 2:** Multivariate analysis of the predictors of time to the first kidney disease-related hospitalization or death using a Cox proportional hazards model in ESRD patients on regular hemodialysis.

Covariate	Estimate	Standard Error	Wald *z* Test	*p* Value	Hazard Ratio	95% Confidence Interval
Hospitalization frequency within 1 year before blood sampling	0.3345	0.0741	4.5153	<0.0001	1.3972	1.2084–1.6156
45.0 years < age ≤ 56.9 years	1.2961	0.3834	3.3806	0.0007	3.6549	1.7240–7.7484
Body mass index ≤ 22.56 kg/m^2^	1.1039	0.3986	2.7694	0.0056	3.0158	1.3808–6.5870
Cholesterol ≤ 130.1 mg/dL or > 209.4 mg/dL	0.9246	0.3500	2.6413	0.0083	2.5207	1.2693–5.0059
Na ≤ 139.52 mmol/L	1.5014	0.5811	2.5839	0.0098	4.4879	1.4370–14.0165
ALT ≤ 6.0 U/L or ALT > 15.3 U/L	1.2440	0.3175	3.9181	<0.0001	3.4695	1.8621–6.4643
Platelet ≤ 115.44 × 10^3^/μL	1.2076	0.5549	2.1764	0.0295	3.3456	1.1276–9.9261
Phosphate ≤ 3.47 mg/dL or > 6.25 mg/dL	0.9543	0.3209	2.9741	0.0029	2.5969	1.3846–4.8707
Alkaline phosphate > 64.31 U/L	1.3197	0.3835	3.4410	0.0006	3.7422	1.7648–7.9355
TIBC (µg/dL)	−0.0125	0.0041	−3.0321	0.0024	0.9876	0.9797–0.9956
Transferrin saturation < 24.96% or > 51.27%	1.2766	0.3846	3.3198	0.0009	3.5846	1.6869–7.6169
hs-CRP > 2.21 mg/L	1.1040	0.5873	1.8799	0.0601 *	3.0163	0.9541–9.5362
IL-18 > 69.05 pg/mL	1.2166	0.5010	2.4283	0.0152	3.3757	1.2644–9.0122
log(SOD3) ≤ 4.72 and 1.11 < Kt/V ≤ 1.87	−1.2982	0.4485	−2.8946	0.0038	0.2730	0.1133–0.6576
log(SOD3) > 4.72 and 1.11 < Kt/V ≤ 1.87	−0.7395	0.4638	−1.5946	0.1108 *	0.4773	0.1923–1.1847

Abbreviations: hs-CRP = high-sensitivity C-reactive protein, IL = interleukin, SOD3 = superoxide dismutase 3, and TIBC = total iron binding capacity. The goodness-of-fit (GOF) measure, concordance = 0.8462 (se = 0.0252) > 0.7 and adjusted generalized *R*^2^ = 0.4887 > 0.3 indicated a good fit. The covariates considered in the stepwise variable selection procedure with the significance level for entry (SLE) and the significance level for staying (SLS) both set to 0.15 during the regression analysis are listed in Table 1. Model-fitting techniques for regression diagnostics (e.g., verification of proportional hazards assumption, residual analysis, detection of influential cases, and check for multicollinearity) were used to assure the quality of the analytical result. * 0.05 < *p* ≤ 0.10: marginally or borderline significant; 0.10 < *p* ≤ 0.15: Barely significant.

## Data Availability

The data presented in this study are available in the article.
